# Population of Greater New York

**Published:** 1896-06

**Authors:** 


### Population of Greater New York.

It is estimated by Dr. Roger S. Tracy, of the New York City Board of Health, that the number of persons living within the limits of "greater New York" is 3,195,059.

